# Advanced Diagnostic Technology of Volatile Organic Compounds Real Time analysis Analysis From Exhaled Breath of Gastric Cancer Patients Using Proton-Transfer-Reaction Time-of-Flight Mass Spectrometry

**DOI:** 10.3389/fonc.2021.560591

**Published:** 2021-04-29

**Authors:** Yoon Ju Jung, Ho Seok Seo, Ji Hyun Kim, Kyo Young Song, Cho Hyun Park, Han Hong Lee

**Affiliations:** Division of Gastrointestinal Surgery, Department of Surgery, Catholic Cancer Research Institute, College of Medicine, The Catholic University of Korea, Seoul, South Korea

**Keywords:** diagnosis, volatile organic compound, stomach neoplasm, breath analysis, screening

## Abstract

**Background:**

Screening endoscopy is considered to be the most accurate tool for early detection of gastric cancer, but it is both invasive and costly. It is therefore essential to develop cost-effective and non-invasive diagnostic tools for gastric cancer. The aim of this study is to investigate the presence of certain volatile organic compounds (VOCs) associated with gastric cancer and to survey the usefulness of VOCs as screening tools of gastric cancer.

**Methods:**

The present study was conducted prospectively to identify the relationship between gastric cancer and specific VOCs quantified by mass spectrometry. Exhaled breath samples from a total of 43 participants were analysed. This study was approved by the Institutional Review Board of the College of Medicine, Catholic University of Korea (KC16TISI0598), and registered to clinical research information service (KCT0004356).

**Results:**

Nine VOCs differed significantly between the control and cancer patient groups. When participants were divided into control, early gastric cancer (EGC), and advanced gastric cancer (AGC) groups, seven VOCs remained significantly different. Of these, four (propanal, aceticamide, isoprene and 1,3 propanediol) showed gradual increases as cancer advanced, from normal control to EGC to AGC. In receiver operating characteristic curves for these four VOCs, the area under the curve for gastric cancer prediction was highest (0.842) when more than two VOCs were present.

**Conclusions:**

The present study offers potential directions for non-invasive gastric cancer screening, and may inspire advanced diagnostic technologies in the era of smart home healthcare. However, despite the high accuracy, cancer-specific VOCs from several studies on different populations, and analytic methods show inconsistency, it is necessary to establish standards for each analytical method, and to validate on each population.

## Introduction

Gastric cancer produces no symptoms until it is well-advanced; early diagnosis and a good prognosis are difficult to achieve without screening by endoscopy. As the rate of early detection *via* endoscopy has increased in East Asian countries such as Japan and Korea, more than 60% of gastric cancers diagnosed in the past 10 years have been early stage (1). Five-year overall survival of patients in this group has been close to 90% (1, 2). It is essential to develop additional effective diagnostic tools for gastric cancer that are minimally invasive, convenient, and cost-effective.

**Figure 1:**
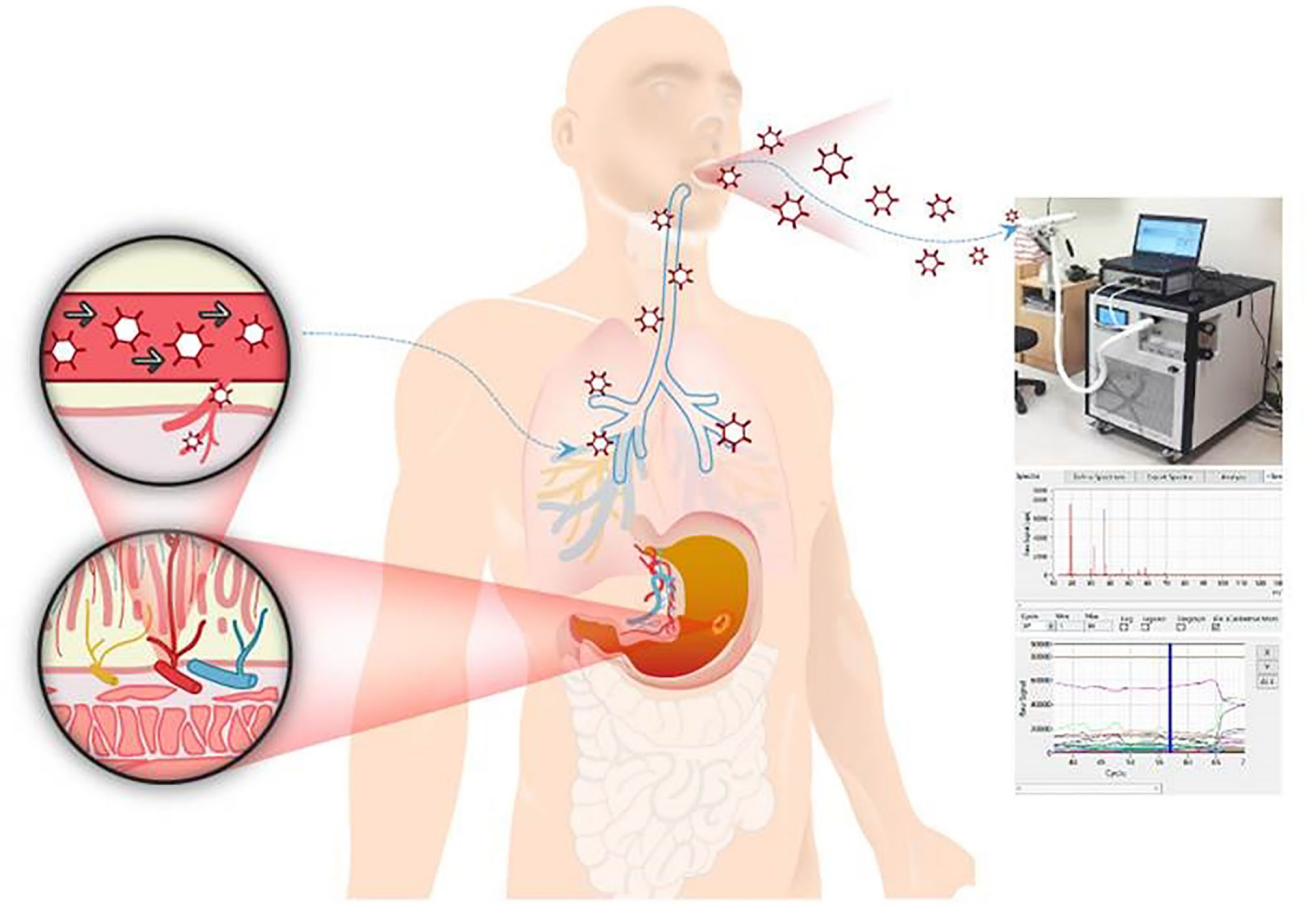
The analysis of volatile organic compounds (VOCs) from exhaled breath *via* Proton-transfer-reaction time-of-flight mass spectrometry (PTR-TOF-MS). This analysis is based on the concept that VOCs are not derived directly from the lung or GI organs but from the metabolic origin through the blood circulating system. Participants’ exhaled breaths samples were repeatedly collected through the mouth inlet of PTR-TOF-MS.

While research on the exhaled breath dates from the time of Hippocrates, who described in his treatise on breath aroma and disease, its development has recently begun to accelerate (3, 4). Since the 1970s, 250–280 different volatile organic compounds (VOCs) have been identified in human urine and exhaled breath (5). Studies on canine olfaction, in which diseases were diagnosed after exposing dogs to human breath, and on cancer diagnosis using electronic nose detection systems, then followed (6). With these developments, VOCs have become a topic of interest in a wide range of medical fields, and have been examined in the context of cardiovascular disease, oncology, neurodegenerative disease, respiratory disease, gastrointestinal disease and diabetes (7). Based on the concept that VOCs are not derived directly from the lung or gastrointestinal organs but from the metabolic origin through the blood circulating system, VOCs associated with solid tumours such as lung, bladder, pancreas, breast or gastric cancers have been proposed in a number of studies (**Figure 1**) (6, 8–10).

Breath analysis has been used in the diagnosis of gastrointestinal disease, in the carbon-13 urea breath test for *Helicobacter pylori* infection, and in hydrogen lactose breath tests to detect small bowel bacterial overgrowth (11, 12). Recently, the developments of analytic tools that enable the collection of large amounts of quantitative and qualitative data are accelerating research into non-invasive cancer diagnosis. The first study in Asia was conducted in China, containing 37 gastric cancer patients which reported that 5 VOCs from breath analysis *via* gas chromatography–mass spectrometry could differentiate cancer with 90% accuracy (13). In this trial, the alveolar breath from the end of the exhalation was filled into a 4L Tedlar bag. Otherwise we here, introduce real time analysis using Proton-transfer-reaction time-of-flight mass spectrometry (PTR-TOF-MS) with direct breath collection system through mouth inlet (13). The present study was conducted prospectively to identify the relationship between gastric cancer and specific VOCs quantified by mass spectrometry, and to confirm the utility of VOCs as tumour markers.

## Materials and Methods

### Study Population

Exhaled breath was collected from a total of 48 participants at Seoul St. Mary’s Hospital between July 2017 and June 2018. Patients diagnosed with gastric adenocarcinoma and scheduled for curative surgery were enrolled; patients with other concurrent cancers or benign gastrointestinal disease, such as inflammatory bowel disease, irritable bowel syndrome, or celiac disease, were excluded (n = 28). Early gastric cancer (EGC) was defined as tumour limited to the gastric mucosa and/or submucosa, regardless of lymph node metastasis. The control group comprised 19 individuals who underwent endoscopic screening and were confirmed to have no neoplastic lesions. Of 48 participants, data from exhaled breath samples were available and analysed for 43 (26 cancer patients and 17 controls). This study was approved by the Institutional Review Board of the College of Medicine, Catholic University of Korea (KC16TISI0598). Patient records were anonymised and de-identified before analysis.

### Exhaled Breath Sampling

Breaths sample and analysis was performed in a separate endoscopy preparation room, and all participants were exposed to the same test environment, temperature, and humidity conditions, without any medical intervention. All participants were fasted for at least 8 h before sampling, and took a deep breath as a test exercise before blowing through a mouth inlet. After a single deep nasal inhalation, exhaled breath was sampled three times. From among the three exhalation peaks, that which was most clearly distinguishable from the ambient gas background was subjected to VOC analysis, conducted in real time through PTR-TOF-MS (**Supplemental Figure 1**).

### VOC Analysis by PTR-TOF-MS

PTR-TOF-MS, a very sensitive method for real-time gas analysis, was used to identify and measure VOCs (14). PTR-TOF-MS consists of an ion source and a drift tube. Reagent ions (H_3_O^+)^ generated by the ion source are injected into the drift tube. When the sample gas containing analyte VOCs is introduced into the drift tube, VOCs can undergo proton transfer reactions with H_3_O^+^, if the proton affinity (PA) values of the trace VOCs exceeds that of H_2_O (PA ¼ 691 kJ/mol), as shown by the following equation: H_3_O^+^ + VOCs/VOCsH^+^ + H_2_O.

All PTR-TOF-MS intensity signals of product ions are given in counts per second (cps), which is proportional to the concentration of VOCs in human breath. VOCs in ambient air will enter the body with the breath; it is essential to exclude this environmental contamination. Ambient air was analysed first, and we compared ambient and exhaled breath to precisely quantify the VOCs. Data were processed using the following formula: A = B – C (A: target compound cps, B: cps of exhaled air, C: cps of ambient air) (**Supplemental Figure 1**).

We initially screened for a total of 57 substances such as aldehydes, alcohols, ketones, and aromatic hydrocarbons including fatty acids, which are in the gastrointestinal cancer-related categories in the previous VOC analysis references (6, 10, 13). Among them, 41 substances detected in more than 80% of the participants were analysed. A list of the screened VOC substances is attached to **Supplement Figure 1C**.

### Statistical Analysis

Chi square or Fisher’s exact tests were used to evaluate between-group differences in categorical variables. Goodness of fit was assessed by calculating the area under the curve (AUC) of the receiver-operating characteristic (ROC) curve, and the optimal cut-offs value was determined using the Youden index. Pearson correlation coefficients among VOCs were examined. All statistical analyses were performed using SPSS version 22.0 (SPSS, Chicago, IL, USA), and a p value < 0.05 was deemed to indicate statistical significance.

## Results

### Patient Characteristics

The clinicopathological characteristics of the participants are shown in **Supplemental Table 1**. The mean age of the cancer patients was older than it of control group (59.2 vs 46.1 years, *P* < 0.001). Among 26 cancer patients, 14 (53.8%) reported no alcohol consumption and 12 (46.2%) had no history of smoking. Smoking history was significantly lower in control group (None; 82.4% in control group, *P *= 0.034). Among cancer patients, 18 (69.2%) were revealed to have *Helicobacter pylori* infection of the gastric mucosa in tissue examination by rapid urease test, polymerase chain reaction, or Warthin-Starry silver test. For control group, in spite of that only 5 participants underwent Helicobactor pylori test, 2 were revealed that had infection, which was not significantly different from cancer patients (69.2% vs 40%, *P *= 0.360). In cancer group, 5 patients (19.2%) were taking proton-pump-inhibitors during the study period, and it was not significantly different from control group (17.6% in control, *P *= 0.656). After radical gastrectomy, 14 patients (53.8%) were diagnosed with EGC, and 12 (46.2%) with advanced gastric cancer (AGC). Sixteen patients (61.5%) had no lymph node metastasis (**Supplemental Table 1**).

### VOCs to Identify Gastric Cancer

Nine VOCs were significantly different between the control and cancer groups. Four VOCs were significantly higher in cancer patients than in normal controls (propanal, aceticamide, isoprene, and 1,3-propanediol, all *P* < 0.05), and five were significantly lower in cancer patients than in normal controls (ethylene, methyl isobutyl ketone, acetic acid, m-tolualdehyde, and 1,3,5-trimethylbenzene, all *P* < 0.05; **Supplement Table 2**).

**Figure 2:**
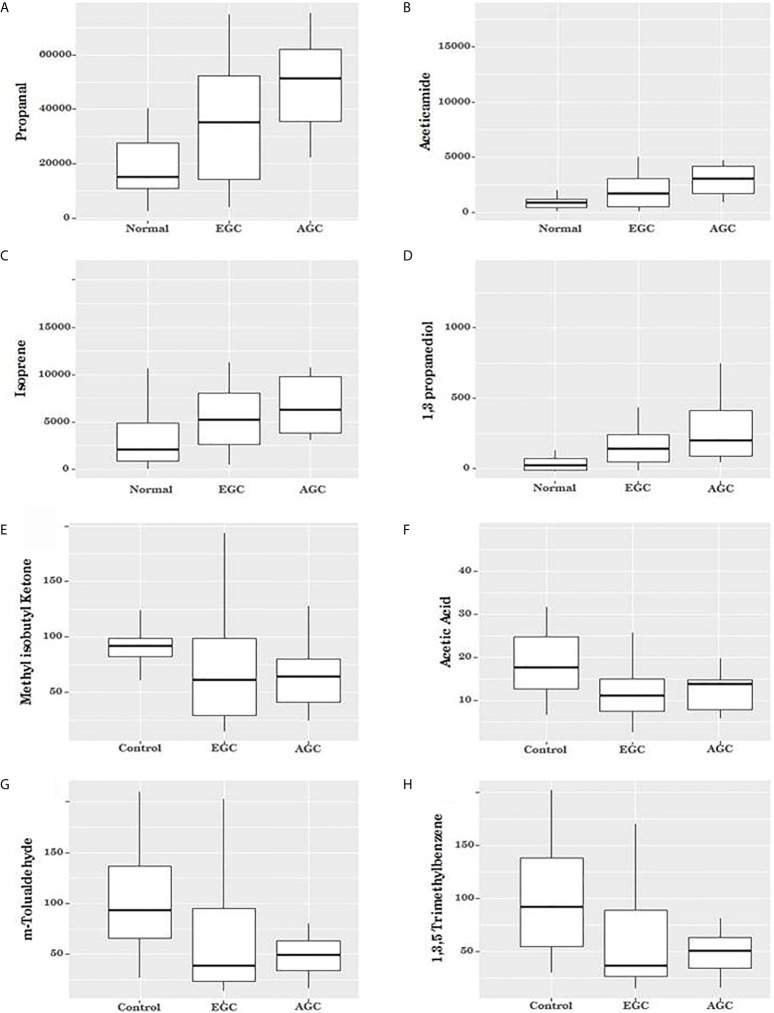
Box plot for the volatile organic compounds (VOCs) according to cancer status. **(A–D)** When patients were grouped according to cancer stage, four VOCs were seen to gradually increase as cancer advanced (Propanal, Aceticamide, Isoprene, and 1,3 propanediol, respectively (*P* = 0.003, *P *= 0.007, *P* = 0.020 and *P* = 0.025, respectively). **(E, F)** The two the VOCs showed no significant differences among the three groups (methyl isobutyl ketone, *P* = 0.998 and acetic acid, *P* = 0.050). **(G, H)** Two of the VOCs that were lower in cancer patients than in controls showed significant differences among the three groups, but these were not correlated with cancer stage (m-tolualdehyde, *P* = 0.021, and 1, 3, 5-trimethylbenzene, *P* = 0.016).

When patients were grouped according to cancer stage, four VOCs were seen to gradually increase as cancer advanced (early cancer > control, advanced > early, all *P* < 0.05; **Table 1** and **Figure 2**). Two of the VOCs that were lower in cancer patients than in controls also showed significant differences among the three groups, but these were not correlated with cancer stage (m-tolualdehyde, *P *= 0.021, and 1,3,5-trimethylbenzene, *P *= 0.016; **Table 1** and **Figure 2**). The remaining three VOCs showed no significant differences among the three groups (ethylene, methyl isobutyl ketone, and acetic acid, *P *= 0.137, *P *= 0.998, and *P *= 0.050, respectively; **Table 1** and **Figure 2**).

**Table 1:** Counts per second of the VOCs according to the Cancer Stages.

	Normal (N=17)	EGC (N=16)	AGC (N=10)	P value
**Propanal**	15127.0 [10963.7-27682.2]	35272.2 [13154.2-54627.8]	51243.3 [35154.9-62143.4]	0.003
**Aceticamide**	942.5 [509.1-1243.0]	1801.5 [558.0-3421.6]	3070.6 [1734.2-4207.8]	0.007
**Isoprene**	2096.0 [862.8-4857.4]	5232.7 [2457.1-8432.7]	6179.1 [3678.3-10149.3]	0.020
**1,3-propanediol**	37.9 [6.0-89.8]	159.0 [54.3-294.5]	216.7 95.3-429.9]	0.025
**Ethylene**	658.0 [518.0-1439.0]	413.5 [160.5-692.0]	569.0 [238.0-610.0]	0.137
**Methyl isobutyl ketone**	92.0 [82.0-99.0]	61.5 [28.0-102.0]	64.5 [36.0-83.0]	0.098
**Acetic acid**	18.0 [13.0-25.0]	11.5 [7.5-15.5]	14.0 [8.0-15.0]	0.050
**m-Tolualdehyde**	95.0 [67.0-138.0]	40.0 [24.5-104.0]	49.5 [33.0-65.0]	0.021
**1,3,5-trimethylbenzene**	92.0 [54.0-138.0]	36.0 [25.5-96.5]	50.5 [32.0-66.0]	0.016

The median values were presented and the numbers in square brackets mean ranges. Chi square test was used to evaluate between-group differences in categorical variables and a p value < 0.05 was deemed to indicate statistical significance. EGC, Early gastric cancer; AGC, Advanced gastric cancer.

### Receiver Operating Characteristic Curves for Gastric Cancer Prediction

**Figure 3:**
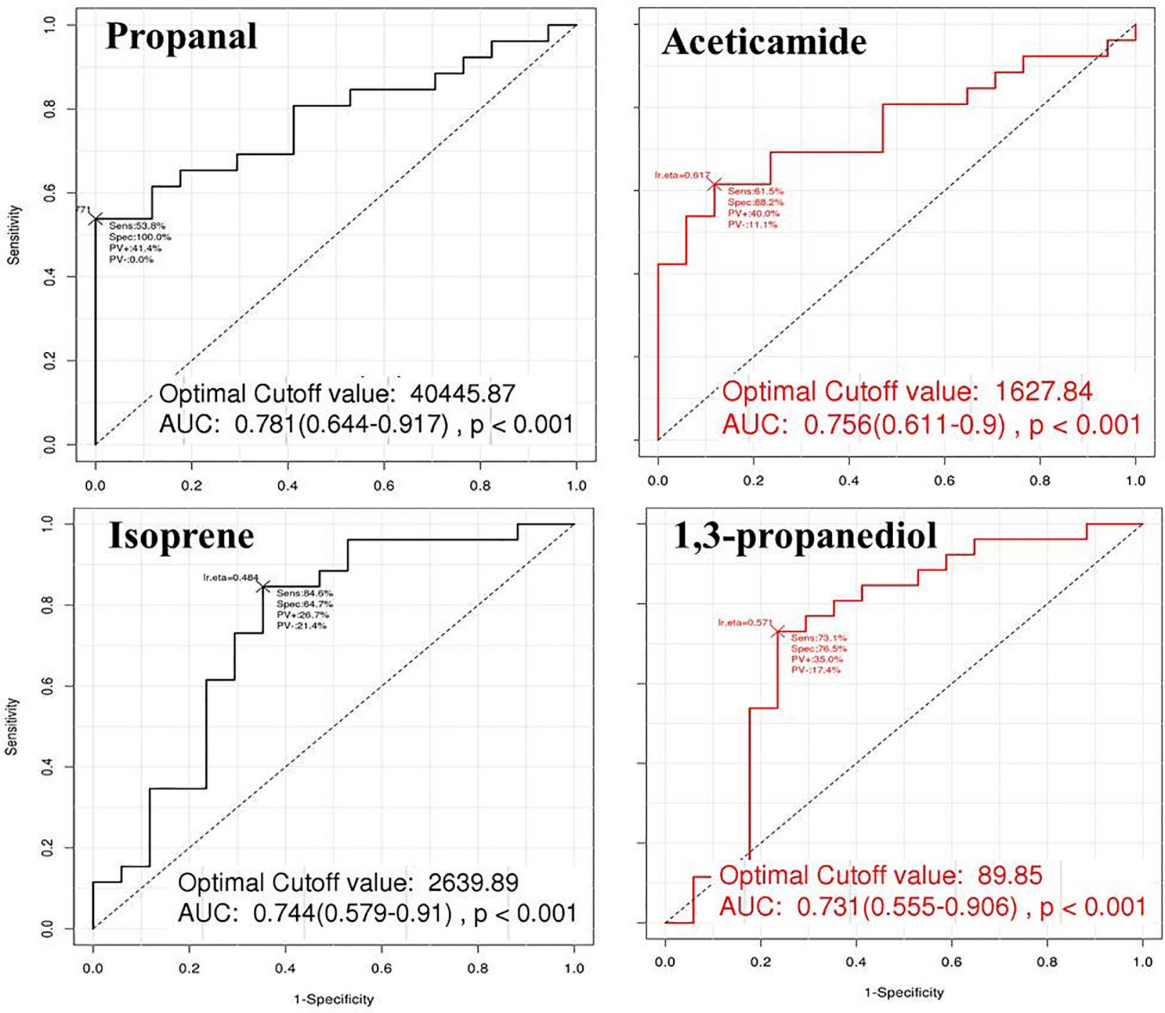
The Receiver operating characteristic (ROC) curves for the effectiveness of volatile organic compounds (VOCs) to predict gastric cancer. The ROC curves were constructed for the four VOCs that increased with cancer stage. The areas under the curve (AUC) for gastric cancer prediction ranged from 0.731 to 0.781 among the VOCs. Propanal showed highest level of AUC of 0.781 with cutoff value of 40 445.87 cps.

The ROC curves were constructed for the four VOCs that increased with cancer stage. The AUC for gastric cancer prediction ranged from 73% to 78% among the VOCs (**Table 2** and **Figure 3**). Cut-off levels were determined by the point on each curve farthest away from the chance diagonal, to maximise sensitivity and specificity.

**Table 2:** Accuracy, sensitivity and specificity for the Volatile organic compounds for the gastric cancer prediction model.

	Accuracy (AUC)	Sensitivity	Specificity	Negative predictive value	Positive predictive value
**Propanal**	78.1%	53.8%	100.0%	58.6%	100%
**Aceticamide**	75.6%	61.5%	88.2%	60.0%	88.9%
**Isoprene**	74.4%	84.6%	64.7%	64.7%	78.6%
**1,3-propanediol**	73.1%	73.1%	76.5%	65.0%	82.6%
**Including** 4 VOCs*	84.2%	61.5%	94.1%	61.5%	94.1%

*When a VOC level was higher than its cut-off value, the VOC was defined as positive and a new Receiver Operating Characteristic curve was constructed based on the positivity status of the four VOCs. AUC, Area under curve.

**Figure 4:**
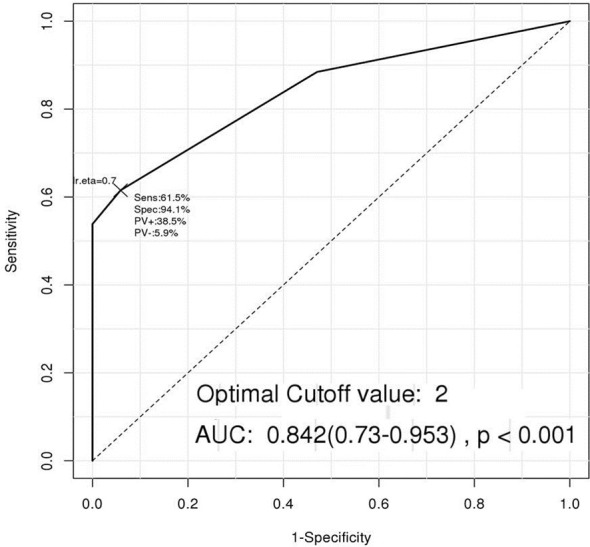
The Receiver operating characteristic (ROC) curve of a new predicting model including the four volatile organic compounds (VOCs). When a VOC level was higher than its cut-off value, the VOC was defined as positive, and a new ROC curve was constructed based on the positivity status of the four VOCs. This model showed the highest AUC of 0.842 with 61% sensitivity and 94% specificity when more than two VOCs were positive.

We performed additional analyses to investigate whether diagnostic accuracy could be improved by combining these four VOCs, rather than basing predictions on single cut-offs for each. When a VOC level was higher than its cut-off value, the sample was defined as positive. In a ROC curve constructed based on the positivity status of the four VOCs, the AUC was highest, at 0.842, when more than two VOCs were positive. This model showed the highest accuracy, with 61% sensitivity and 94% specificity (**Table 2** and **Figure 4**).

### Correlation Analysis Among VOCs

Pearson correlation analysis was conducted to determine whether any up- or down-regulated VOCs were consistently correlated (**Supplemental Figure 2**). Propanal was positively or negatively correlated with all VOCs (all *P* < 0.05) except methyl isobutyl ketone and acetic acid (**Supplemental Figure 2**).

## Discussion

Endoscopic surveillance is the most sensitive and effective screening method for gastric cancer and endoscopic screening has been widely used for early detection, especially in East Asia where the incidence of gastric cancer is high. However, endoscopic biopsy is invasive and many people are subjected to unnecessary examinations when it is used as a screening tool. Moreover, in Western countries, where the medical costs related to endoscopic diagnosis and treatment of upper gastrointestinal disease have been estimated at up to $88 000, endoscopic screening for the general population is considered impractical (15). Blood tests and liquid biopsies or the upper gastrointestinal series are less invasive diagnostic modalities with potential, but their accuracy and efficacy remain unsatisfactory (16, 17).

In the present study, there were 9 VOCs which were significantly different between the control and cancer patient groups. And among those, 4 VOCs (propanal, aceticamide, isoprene and 1,3 propanediol) showed gradual increases as cancer advanced, from normal control to EGC to AGC with 84% of accuracy in cancer detection when more than two VOCs were present. However, because of the small sample size, there were some differences in the characteristics such as age and smoking history between the cancer and control groups (**Supplement Table 1**). However, these 9 VOCs were not significantly different according to the age (older than 60 or under) and smoking history (data is not shown). Furthermore, according to the previous reports from the studies on lung cancer patients, VOCs affected by smoking were toluene, benzene, acetonitrile, 2-methyl furan, 2,5-dimethyl furan, 1,3-cyclohexadiene, and 1,3-cyclopentadiene, which does not overlap with the 9 VOCs shown in our results (3, 9). Therefore, the above 9 VOC is likely to be a cancer specific target material.

While VOCs have been studied for several decades, there are few reports of gastric cancer-specific VOCs used for screening (13, 18–20). A recent study on 210 individuals, including 33 gastric adenocarcinoma patients, reported that eight VOCs (decanal, nonanal, phenol, ethyl phenol, methyl phenol, hexanoic acid, heptanal, and butyric acid) could distinguish gastric cancer patients from normal controls, and the accuracy of cancer prediction was almost 90% (19). Another report, on 484 patients, including 99 with gastric cancer, also demonstrated over 90% accuracy in cancer prediction, even based on precancerous lesions such as high-grade intestinal metaplasia, using eight VOCs (2-propenenitrile, furfural, 2-butoxy-ethanol, hexadecane, 4-methyloctane, 1,2,3-tri-methyl-benzene, α-methyl-styrene, and 2-butanone). When they analysed the above VOCs according to sex, age, smoking/alcohol consumption, helicobacter infection, and proton pump inhibitor medication, it was found that the above VOCs were not affected (18).

Most of the previous studies were conducted on European populations; to our knowledge, there is one study conducted in Asia with a high incidence of gastric cancer. This study from Chinese population reported 5 VOCs (2-propenenitrile, 2-butoxy-ethanol, furfural, 6-methyl-5-hepten-2-one, and isoprene) could distinguish gastric cancer with 90% accuracy (13). Comparing our results with those of several other studies, it can be seen that there are three main types of VOCs commonly detected in gastric cancer patients: fatty acids, alcohols, and aldehydes. Although the classifications overlap in some studies of gastrointestinal cancers, including our own, only a few compounds are consistently present, such as isoprene, trimethyl benzene, and propanal (13, 18, 21).

The mechanisms underlying specific VOCs detected in cancer patients have not been elucidated, but there are some hypotheses regarding the generation of these compounds. Fatty acids are hydrocarbons that undergo lipid peroxidation due to oxidative stress in the body. As the unsaturated fatty acids in human body are composed with unbranched types, branched fatty acids cannot be derived from lipid peroxidation through normal metabolism (7). Accordingly, high concentrations of branched fatty acids may reflect pathologic conditions. Indeed, some branched hydrocarbons have been found in the exhaled breath of patients with cancers, including gastric adenocarcinoma (8, 10, 21).

Isoprene, an unbranched hydrocarbon that is among the most abundant hydrocarbons measurable in exhaled breath, is derived from the mevalonate pathway underlying cholesterol synthesis (22). It was reported that the breath of healthy, relaxed volunteers contained very low concentrations of isoprene, with a median level of 100 ppb (9). In our study, the normal control group exhaled even lower levels of isoprene (median of 49 ppb vs. 226 ppb for the cancer group, *P* = 0.001, data not shown). A high concentration of isoprene in breath analysis has been thought to reflect psychological stress (7, 23, 24). However, recently it was shown to be associated with immune system function in lung cancer patients (25). In addition to lung cancer patients, other cancer patients may show high levels of isoprene in exhaled breath, although detailed studies of immune reactions to carcinogenesis are needed to clarify this relationship.

Primary alcohols can be oxidised to aldehydes by a variety of enzymes during normal metabolism, including alcohol dehydrogenase and cytochrome P450 2E1 (CYP2E1). During carcinogenesis, alcohols and aldehydes can form through lipid peroxidation *via* cytochrome P450 (26). When the alcohol level increases, reactive oxygen species (ROS) can be generated from oxidizing the alcohol to acetaldehyde by CYP2E1 (27, 28). The P450 is known to be closely related to carcinogenesis. This is firstly based on the fact that ROS produced in the process mediated by CYP2E1 is highly detected in several cancer cells. The ROS can be generated by oncogene activation, metabolic alterations or macrophage infiltration or hypoxia/reoxygenation processes in tissues, such as DNA damage, autophagy, and angiogenesis, resulting tumour formation or progression (27). In addition, several papers have shown results that exhaled aldehyde levels are not affected by age and sex (29–32). Second, it is hypothesized that more aldehydes will be produced because the cancer cell membrane contains an abundant amount of saturated lipids than that of normal cells (27). As this occurs mainly in the liver, alcohols and aldehydes in exhaled breath are regarded as being derived from the systemic circulation, which involves liver metabolism followed by gas exchange at the lungs, and not from the upper GI tract (**Figure 1**). And aldehydes have low solubility in blood, so they are expelled through the exhaled breath immediately after a few minutes of production in body tissue. Hence, breath alcohol and aldehydes may reflect systemic metabolic reactions during carcinogenesis (18).

Breath analysis can be one of the best methods in terms of developing a non-invasive diagnostic tool for gastric cancer. Since the VOCs are basically thought to be generated from the metabolic reaction of systemic or peritumoral environment, flows through the blood stream, and finally excreted into the breath, it might be considered that it might be helpful to analyze VOCs from patients’ blood or tissue medium to validate the breath analysis results and prove the producing mechanism. However, according to a review article about cancer related VOCs, published in 2019, out of 668 studies using breath, fluids (blood, urine, saliva, and bile), culture medium, tissue, the number of studies using breath was 266 (39.8%), followed by 151 of cell culture media use (22.6%), 85 of tissue use (12.7%), and only 25 of blood use (3.7%) (27). This might be because blood analysis is considered as invasive and time/cost consuming for VOC analysis, and additionally there are too many factors that may cause biases such as temperature, pH, pK, and ionic contents of the sample (27, 33). And the most important difference between breath analysis compared to blood analysis is that it is non-invasive and allows multiple tests to be performed quickly and easily with low costs. Furthermore, to compare with endoscopic examination, which is currently widely used in Asia as the most accurate screening tool, blood analysis consuming massive costs to control environments of the samples to avoid biases, has little advantages in terms of invasive and cost-effectiveness.

On the other hand, urine, feces, or saliva sample can be a good research field that should be included to VOC analysis as well as microbiome-related research that can affect gastric cancer development in the future. Although *in vitro* experiments using cancer cells of patients’ tissues might have a strength in identification of mechanism directly with easy interpretation of VOC producing in tumors, it is difficult to represent human body process, consists with full of complex reactions. Therefore, conducting well-designed comprehensive study that includes *in vitro* experiments and VOC analysis using patients’ fluids samples to compare breath VOCs for gastric cancer screening should be required in the future.

The strength of this study lay in the methodology. As a real-time direct mass spectrometry method, the sensitivity of PTR-MS is higher than that of selected ion flow tube mass spectrometry (SIFT-MS) and it generates different precursor ions $\left({\text{H}}_{3}{\text{O}}^{+},{\text{O}}_{2}^{+},\text{and}\;\text{N}{\text{O}}^{+}\right)$ (34). PTR-MS has been developed for use with time-of-flight (TOF) instruments, one of the newest types of breath VOC detection technology (34). Furthermore, we collected exhaled samples directly through a mouth inlet to the instrument, rather than in transported sample bags, which minimised sample contamination and allowed for repetitive sampling without extra costs. In this study, participants blew the exhalation 3 times and the most distinguishable peak was used for analysis. In addition, the VOC levels measured in cps unit could be more accurate results which can compensate for the difference in exhaled breath volume or contaminated ambient gas than parts per billion (ppb) units measurement.

There were limitations to our study. Firstly, it included a small numbers of patients drawn from a single centre. Validation studies with pre-set cut-off values for VOCs to detect gastric cancer will be required. And what we should note when dealing with the results on specific VOCs discovery is that there could be a chance of false positivity which can occur when a large number of variables were investigated. This is called ‘Voodoo correlations’ that can be resulted as a statistically true correlation, which appear coincidentally in the large number of variables (7, 35). To reduce this accidental detection of the cancer-related VOCs, the initial screening was performed on the list of subjected VOCs subject, focusing on substances that have been measured among the previously published literature, nonetheless, the chance of such false positivity still exists. In order to compensate for this point, studies on a larger cohort with validation of the mechanisms should be accompanied in the future. The significance of our study as the first step in such a process is to identify the existence of a potential VOCs and to be able to plan a comprehensive study for this, in addition, our results is supported by the discovery of substances in similar categories in other cancer related reports. And a common data collection from the exhaled breath samples in large-scale databases such as Human Metabolome Database or CanSAR will advance this research to the next step of true implantation to the clinical fields (36, 37).

Secondly, we did not determine whether the VOCs distinguish gastric cancer from other malignancies of the gastrointestinal tract or elsewhere. To identify VOCs specific to certain cancers, large cohort studies including various type of cancers and appropriate diagnostic tools will be needed. Thirdly, postoperative changes in the VOCs of cancer patients were not investigated, due to differing clinical courses and follow-up schedules. Fourthly, 8 hours of fasting time before the breath sampling for VOC analysis was considered as the optimal time for gastric emptying without remaining food as much as possible. Since the endoscopic findings which were taken immediately after the breath sampling of all the participants didn’t show any remnant gastric contents, it is thought to be the effect on food diet could be minimized. However, further metabolic reactions that might occur after absorption and digestion of food intake itself, the proper fasting time has not been determined, so further studies are needed to reveal these possible confounding factors. Finally, although we noted some significant quantitative correlations among VOCs, we lack evidence to explain the possible mechanisms underlying these relationships. And many studies and review papers published since now have presented explanations and hypotheses for the causes of cancer related VOCs by category, but the mechanism for each substance itself has not been revealed. Therefore, further studies to reveal the metabolic underpinnings of exhaled VOCs are needed for a comprehensive understanding of the interactions involved. Furthermore, one important issue to be solved in cancer-related VOC studies is that there are no standards by each analytical device, methods, or unit. In addition, it is necessary to investigate other metabolic factors, such as staple food, race, or gut microbiome, which can affect VOC composition and to be compensated. Therefore, future VOC research should be conducted and validated by each population, race, and generation. This might be the reason for the inconsistency between Chinese and our results.

The accuracy of VOC detection as a screening modality for gastric cancer has been confirmed to be greater than 80%, in this and other studies, which is promising for future work. Regarding endoscopy, given the cost of the instruments, time required for the procedure, its invasiveness, and the need for specialists to be involved in every examination, the development of other screening tools that can be easily and repeatedly employed is necessary. Detection of specific VOCs is very easy and sampling can be conducted repeatedly. Thus, it provides speed and convenience, and allows for handling of a large number of patient samples simultaneously. Thus, VOC detection techniques could play an important role in screening prior to endoscopy as a filter, and reduce the number of unnecessary invasive examinations.

We conclude that it has significant potential for non-invasive cancer screening, and may inspire future cancer diagnostic technologies in the era of smart home healthcare. Further studies investigating both the reproducibility of VOC as a diagnostic tool and the relevant mechanisms underlying their generation in the breath are required.

## Data Availability Statement 

The raw data supporting the conclusions of this article will be made available by the authors, without undue reservation.

## Ethics Statement 

The studies involving human participants were reviewed and approved by the Institutional Review Board of the College of Medicine, Catholic University of Korea (KC16TISI0598). The patients/participants provided their written informed consent to participate in this study.

## Author Contributions

YJ and KS, and HL conceived and designed the study. YJ wrote the manuscript and performed data analysis. HS and JK were responsible for data collection and reviewing data analysis. CP and HL reviewed the manuscript and provided feedback. All authors contributed to the article and approved the submitted version. 

## Funding

The authors declare that they have no conflict of interest. And this research was supported by grants from the National Research Foundation of Korea (grant nos. 2018R1D1A1B07045486, 2020R1A2C1012007, and 2020R1I1A1A01072547).

## Conflict of Interest

The authors declare that the research was conducted in the absence of any commercial or financial relationships that could be construed as a potential conflict of interest.
