# Supplementary material for: Advanced Diagnostic Technology of Volatile Organic Compounds Real Time analysis Analysis From Exhaled Breath of Gastric Cancer Patients Using Proton-Transfer-Reaction Time-of-Flight Mass Spectrometry
Source: Front Oncol. 2021 Apr 29;11:560591. doi: 10.3389/fonc.2021.560591 (PMC8116791; doi:10.3389/fonc.2021.560591)
Supplement: Supplementary file 1 [file DataSheet_1.docx]

**Supplemental information**

**Supplemental Figure 1**. Data collection and analysis of VOCs from the exhaled breath. **A;** Collection of patients’ exhaled breaths through the mouth inlet of PTR-TOF-MS. **B**; Analysis of the intensity signals given in counts per second (cps) according to the VOCs. VOC; Volatile organic compound, PTR-TOF-MS; proton-transfer-reaction time-of-flight mass spectrometry.

**Supplemental Figure 2**. Pearson correlation coefficient analysis between VOCs, top line presents r value for each correlations, bottom line with red color presents significant p values.

**Supplemental Figure 1**. Data collection and analysis of VOCs from the exhaled breath. **A;** Collection of patients’ exhaled breaths through the mouth inlet of PTR-TOF-MS. **B**; Analysis of the intensity signals given in counts per second (cps) according to the VOCs. VOC; Volatile organic compound, PTR-TOF-MS; proton-transfer-reaction time-of-flight mass spectrometry.

**C**

**B**

**A**


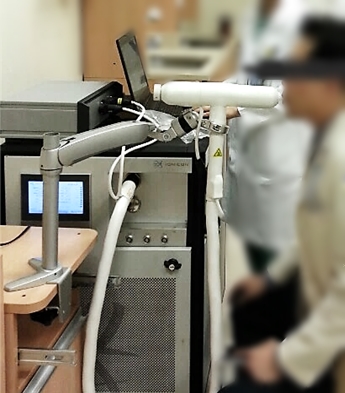

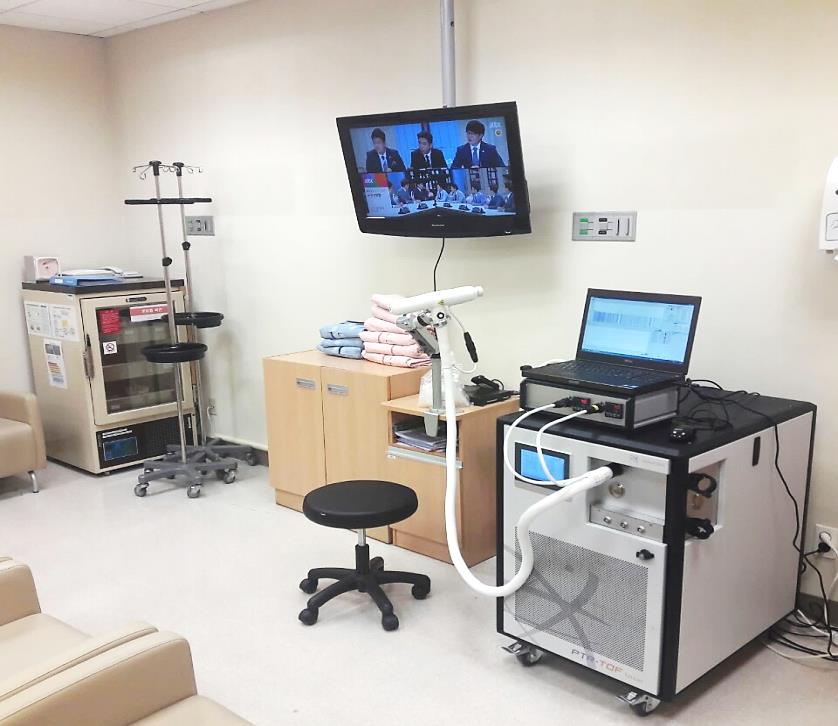

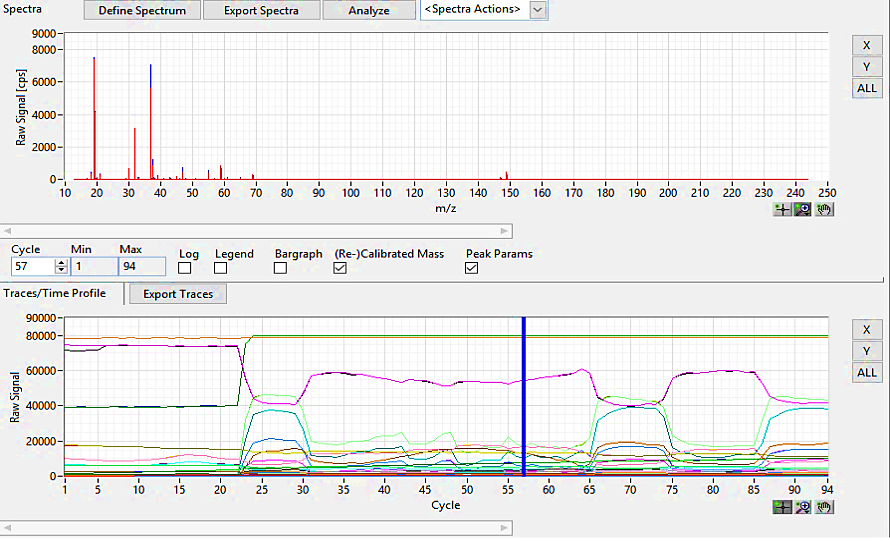


**Supplemental Figure 2**. Pearson correlation coefficient analysis between VOCs, top line presents r value for each correlations, bottom line with red color presents significant p values.


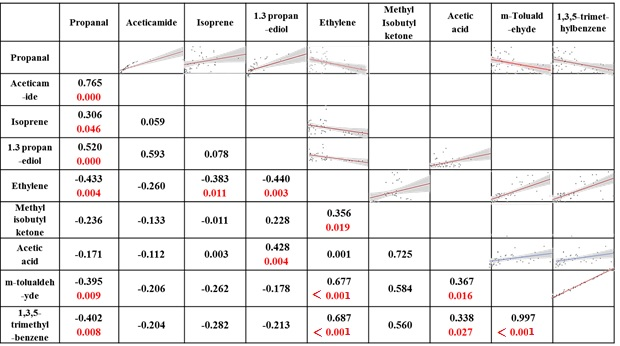


\

**Supplemental Table 1.** Clinicopathological Characteristics of the Participants (Gastric Cancer and Control Groups)

|  |  | **Cancer (N=26)** | **Control (N=17)** | **P value** |
| --- | --- | --- | --- | --- |
| **Age** |  | 59.2 ( ± 9.7) | 46.1 (± 16.5) | 0.001 |
| **Sex (M:F ratio)** |  | 17:9 (65.3%) | 7:10 (41.2%) | 0.079 |
| **Alcohol** | None | 14 (53.8%) | 6 (35.3%) | 0.152 |
|  | Social | 5 (19.2%) | 9 (52.9%) |  |
|  | Heavy | 7 (26.9%) | 2 (11.8%) |  |
| **Smoking** | None | 12 (46.2%) | 14 (82.4%) | 0.034 |
|  | Quit | 11 (42.3%) | 1 (5.9%) |  |
|  | Current | 3 (11.5%) | 2 (11.8%) |  |
| **Helicobactor pylori** | No | 8 (30.8%) | 3 (60%) | 0.360 |
|  | yes | 18 (69.2%) | 2 (40%) |  |
| **PPI medication** | No | 21 (80.8%) | 14 (82.4%) | 0.656 |
|  | yes | 5 (19.2%) | 3 (17.6%) |  |
| **Ulceration** | no | 7 (26.9%) |  |  |
|  | Yes | 19 (73.1%) |  |  |
| **Tumor location** | Upper | 1 (3.8%) |  |  |
|  | Middle | 4 (15.4%) |  |  |
|  | Lower | 12 (46.2%) |  |  |
|  | Whole | 9 (34.6%) |  |  |
| **CEA** |  | 1.3 ± 1.1 |  |  |
| **CA19-9** |  | 28.9 ± 12.9 |  |  |
| **T stage** | T1 | 14 (53.8%) |  |  |
|  | T2 | 4 (15.4%) |  |  |
|  | T3 | 4 (15.4%) |  |  |
|  | T4 | 4 (15.4%) |  |  |
| **N stage** | N0 | 16 (61.5%) |  |  |
|  | N1 | 4 (15.4%) |  |  |
|  | N2 | 2 ( 7.7%) |  |  |
|  | N3a | 3 (11.5%) |  |  |
|  | N3b | 1 ( 3.8%) |  |  |
| **Differentiation** | Differentiated | 10 ( 38.4%) |  |  |
|  | Undifferentiated | 16 (61.6%) |  |  |
| **Lauren** | Intestinal | 13 (50.0%) |  |  |
|  | Diffuse/Mixed | 13 (50.0%) |  |  |
| **Lymphatic invasion** | Negative | 12 (46.2%) |  |  |
|  | Positive | 14 (53.8%) |  |  |
| **Vascular invasion** | Negative | 25 (96.2%) |  |  |
|  | Positive | 1 ( 3.8%) |  |  |
| **Neural invasion** | Negative | 20 (76.9%) |  |  |
|  | Positive | 6 (23.1%) |  |  |
| Data given as numbers (%) and means (±SD). Chi square test was used to evaluate between-group differences in categorical variables and a p value < 0.05 was deemed to indicate statistical significance. PPI; Proton pump inhibitors | | | | |

**Supplemental Table 2**. Counts per second of the Statistically Significant VOCs according to the Cancer Status

|  | **Normal (N=17)** | **Gastric Cancer (N=26)** | **P value** |
| --- | --- | --- | --- |
| **Propanal** | 15127.0 [10963.7-27682.2] | 43452.1 [23548.7-60496.3] | 0.002 |
| **Aceticamide** | 942.5 [509.1-1243.0] | 2317.5 [992.4-3987.3] | 0.004 |
| **Isoprene** | 2096.0 [862.8-4857.4] | 5429.5 [3081.1-9209.3] | 0.007 |
| **1,3-propanediol** | 37.9 [ 6.0-89.8] | 159.0 [82.8-403.9] | 0.012 |
| **Ethylene** | 658.0 [518.0-1439.0] | 544.0 [166.0-678.0] | 0.047 |
| **Methyl isobutyl ketone** | 92.0 [82.0-99.0] | 63.0 [30.0-94.0] | 0.035 |
| **Acetic acid** | 18.0 [13.0-25.0] | 12.0 [ 8.0-15.0] | 0.016 |
| **m-Tolualdehyde** | 95.0 [67.0-138.0] | 43.5 [25.0-73.0] | 0.006 |
| **1,3,5-trimethylbenzene** | 92.0 [54.0-138.0] | 40.5 [26.0-72.0] | 0.004 |

The median values were presented and the numbers in square brackets mean ranges. Chi square test was used to evaluate between-group differences in categorical variables and a *p* value < 0.05 was deemed to indicate statistical significance.
